# Harmonized technical standard test methods for quality evaluation of medical fluorescence endoscopic imaging systems

**DOI:** 10.1186/s42492-024-00184-5

**Published:** 2025-01-10

**Authors:** Bodong Liu, Zhaojun Guo, Pengfei Yang, Jian’an Ye, Kunshan He, Shen Gao, Chongwei Chi, Yu An, Jie Tian

**Affiliations:** 1 Center for Medical Device Evaluation, National Medical Products Administration, Beijing, 100081 China; 2 School of Engineering Medicine and School of Biological Science and Medical Engineering, Beihang University, Beijing, 100191 China; 3 The Key Laboratory of Big Data-Based Precision Medicine, Ministry of Industry and Information Technology of China, Beijing, 100191 China; 4 Key Laboratory of Molecular Imaging, Institute of Automation, Chinese Academy of Sciences, Beijing, 100191 China

**Keywords:** Fluorescence endoscopy, Near-infrared, Image evaluation, Standardization

## Abstract

Fluorescence endoscopy technology utilizes a light source of a specific wavelength to excite the fluorescence signals of biological tissues. This capability is extremely valuable for the early detection and precise diagnosis of pathological changes. Identifying a suitable experimental approach and metric for objectively and quantitatively assessing the imaging quality of fluorescence endoscopy is imperative to enhance the image evaluation criteria of fluorescence imaging technology. In this study, we propose a new set of standards for fluorescence endoscopy technology to evaluate the optical performance and image quality of fluorescence imaging objectively and quantitatively. This comprehensive set of standards encompasses fluorescence test models and imaging quality assessment protocols to ensure that the performance of fluorescence endoscopy systems meets the required standards. In addition, it aims to enhance the accuracy and uniformity of the results by standardizing testing procedures. The formulation of pivotal metrics and testing methodologies is anticipated to facilitate direct quantitative comparisons of the performance of fluorescence endoscopy devices. This advancement is expected to foster the harmonization of clinical and preclinical evaluations using fluorescence endoscopy imaging systems, thereby improving diagnostic precision and efficiency.

## Introduction

Medical endoscopy technology is a seminal advancement in contemporary medicine that has significantly enhanced the efficacy and precision of disease diagnosis. As a high-fidelity, minimally invasive diagnostic instrument, medical endoscopy captures and relays real-time visual data from within the patient’s anatomy to an external display. This is achieved by using a compact camera and light source apparatus, facilitating the unobstructed visual inspection of pathological tissues [1–3]. Consequently, clinicians can gain diagnostic insights that are both visually clear and clinically reliable. Moreover, endoscopes can be integrated with supplementary instruments such as biopsy forceps and therapeutic lasers, thereby enabling the sampling as well as in situ treatment of tissues within the body [4, 5].

The design of an endoscopic system must adhere to several critical requirements, as dictated by the definition of an endoscope. First, because endoscopes must access the human body via either natural orifices or surgical incisions, their design must accommodate diameter constraints and the need for enhanced flexibility. This ensures minimal invasiveness and maneuverability within the cavities of the body. Second, the endoscopic camera system must be complemented by an external light source to illuminate the internal body structures. The design of this light source must ensure that it does not cause thermal injury to the internal tissues, thereby maintaining patient safety. Third, the imaging quality must meet stringent standards to ensure that the clarity and resolution of the endoscopic images approximate the acuity of direct visual inspection. This is crucial for accurate diagnostic assessments and subsequent clinical decisions [6, 7].

Traditional white-light imaging employs xenon or light-emitting diode (LED) light sources, improving upon traditional fiber-optic imaging methods. Existing white-light endoscopes can achieve a resolution of 4K/8K UHD, providing physicians with higher-quality endoscopic images [8–10]. However, traditional white-light imaging relies on the natural reflection of light from the surface of tissues and lacks disease-specific optical characteristics, making it difficult to locate and visualize precancerous lesions [11]. In addition, some diseases may be unevenly distributed within the tissue, forming diffuse and patchy areas, which further hinders disease detection [12].

In contrast, fluorescence endoscopy technology is based on the biochemical properties and related reactions of biological tissues. It irradiates biological tissues by sending a light source of a specific wavelength through a laser (e.g., near-infrared (NIR)); this light source can be absorbed by fluorescent markers in the tissues and then released in the form of fluorescence, which is captured by the endoscopic camera and converted into a digital image for display [13, 14]. There is a significant difference between the fluorescence absorption and the emission spectra of diseased and normal tissues, and fluorescence endoscopy can capture unique fluorescence signals of the diseased area to realize the early detection of lesions and accurate diagnosis [15, 16]. In recent years, this technology has been used extensively in various clinical studies such as precise tumor localization and boundary identification, in vivo 3D visualization of gliomas, and surgical navigation [17–20]. In addition, fluorescence endoscopy technology can provide imaging using a multiple labeling strategy in combination with multiple fluorescent markers [21].

As medical expertise and public health consciousness continue to advance, expectations regarding the quality of fluorescence endoscopic imaging have increased. The design of the fluorescence endoscopic functional supply device and imaging outcomes produced by the fluorescence camera system exert an indirect or a direct influence on the diagnostic process for physicians. Numerous international consensus documents have been established, delineating standardized test methods based on the body mode for various medical imaging technologies. These techniques include magnetic resonance imaging [22], computed tomography [23], and positron emission tomography [24].

In 2009, the International Electrotechnical Commission (IEC) published technical standard IEC 60601-2-18:2009 for medical endoscopic equipment, which covers detailed requirements for the basic safety and essential performance of endoscopic devices [25]. In 2017, the Association of periOperative Registered Nurses (AORN) proposed the AORN MAN-860C-2017 guidelines to assist healthcare professionals in performing care, cleaning, decontamination, and maintenance operations for flexible and rigid endoscopes and related accessories [26]. In 2019, the Pharmaceuticals and Medical Devices Agency approved the first native medical device that uses fluorescence endoscopy technology [27, 28]. In 2020, Kanniyappan et al. [29] published an article on a test method for NIR performance, but the method is not applicable to performance testing of fluorescent endoscopes and uses a phantom with a complex design that limits direct comparisons among different devices and studies. Moreover, the domain of NIR fluorescence (NIRF) endoscopy lacks established technical standards and guidance documents for the quantitative assessment of its imaging capabilities. Consequently, there is an urgent need to develop standardized criteria for evaluating the image quality of endoscopic camera systems. The establishment of unified and standardized protocols for fluorescence endoscopic instruments and the evaluation of fluorescence image quality are of significant practical and clinical importance. This initiative is crucial for enhancing the precision of medical diagnoses and efficacy of clinical applications.

We underscore the importance of developing technical standards for NIRF endoscopy, which are essential for the creation of standardized medical devices in this field. Enhancing the specifications for evaluating the quality of fluorescent images is paramount to ensure consistency and comparability across various devices and operators. It will also enable effective longitudinal comparisons of images that are captured at different time points. By implementing these standardization initiatives, we anticipate a marked enhancement in the performance of fluorescence endoscopy technology and the precision of its clinical applications. These advancements will provide medical professionals with more accurate and dependable diagnostic support. Ultimately, these efforts will benefit patients, elevate the standard of medical care, and improve the overall quality of health services.

## Methods

### Fluorescent endoscopic imaging system

In this study, we employed the commercially available NIRF thoracoscopic imaging system DPM-III-01 provided by Zhuhai Dipu Medical Technology Co., Ltd., to execute the experimental procedure [30, 31]. This system is equipped with real-time triple-window observation capabilities, enabling it to provide white-light (color), fluorescence (monochrome), and overlaid (pseudo-color) images according to the varying demands of different surgical stages. The design flexibility of the DPM-III-01 system allows it to display NIR images, white-light images, or their overlays, and even to present all three simultaneously, offering comprehensive visual information for surgery. This high degree of flexibility and multifunctionality endows the DPM-III-01 system with a crucial role in surgical procedures, significantly enhancing the precision and safety of the operations. During the experimental process, the endoscope camera system software (Version V1.1) was employed to acquire and post-process the camera images.

To select a fluorescent contrast agent, we used an ICG solution at a concentration of 2.5 mg/mL, blended with fetal bovine serum to create a fluorescent solution. ICG, which is recognized for its exceptional safety and fluorescent properties, has received approval from the US Food and Drug Administration, guaranteeing its safety and efficacy in clinical use. Consequently, ICG offered a secure and potent fluorescent labeling alternative for our experimental procedures, thereby reinforcing the dependability and scientific merits of the experiment.

### Fluorescence test mimics

**Fig. 1:**
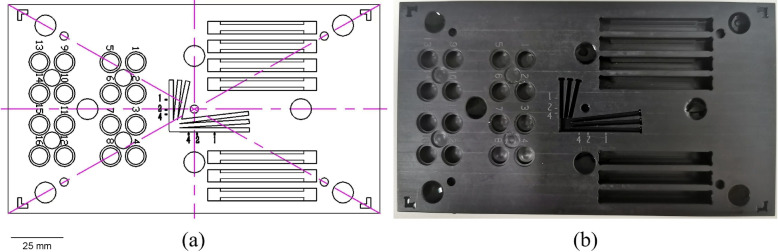
The structure of the imitation body used for the test and the physical drawing. **a** Schematic of the fluorescence test phantom; **b** photograph of the fluorescence test phantom prototype

The fluorescence test simulator was made of black PTFE as the substrate material, and its size and shape were the same as those of the effective area of the transmission resolution test card [NQ/NE-10-50A(0.5X)] required by ISO 12233, i.e., 100 mm $\times$ 178 mm, with a thickness of 20 mm, as shown in Fig. 1. The design of the fluorescence test phantom included specific areas with standard test patterns and holes for capturing fluorescence images using the imaging system. By titrating the appropriate amount of fluorescent contrast agent into the designated test holes and patterns according to the testing protocol, image acquisition for the respective fluorescence testing items could be performed and the resulting images could be evaluated.

**Fig. 2:**
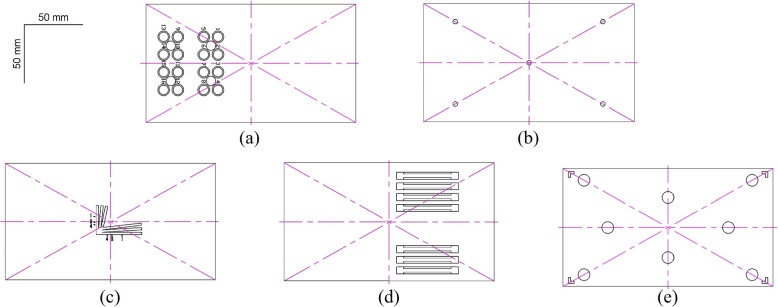
Schematic of the test phantom partitioning. **a** Fluorescence sensitivity, SNR, and fluorescence dynamic range (DR) testing area; **b** fluorescence imaging distortion and fluorescence image brightness uniformity testing area; **c** fluorescence resolution resting area; **d** fluorescence detection depth testing area; **e** fusion accuracy testing area

The partition layout of the test phantom is depicted in Fig. 2, with Fig. 2a showing the evaluation areas for the fluorescence sensitivity, signal-to-noise ratio (SNR), and dynamic response range. This section was composed of 16 numbered circular wells on the left and an interspersed set of four additional circular wells. The 16 numbered circular wells were intended to contain fluorescent contrast agents of different concentrations with dimensions of 8 mm $\times$ 5 mm. To prevent spillage of the contrast agent during the transfer process, which could affect the test by exceeding the upper surface, a concentric recess measuring 10 mm $\times$ 1 mm was also included. Four smaller circular wells were designated for the selection of background signals in the SNR testing, with dimensions of 8 mm $\times$ 2 mm.

Figure 2b depicts the fluorescence resolution testing area, which was composed of the central fork-like pattern shown in Fig. 1. The branches of the pattern (straight grooves) were 2 mm wide and 3 mm deep, with the spacing between adjacent straight grooves increasing gradually from 0 mm to 2 mm without exceeding a slope of 0.1.

Figure 2c shows the fluorescence detection depth testing area, which consisted of the seven different depths of straight slots shown on the right side of Fig. 1, forming the fluorescence detection depth test block. An appropriate slot size was selected based on the diameters of the quartz glass tubes and detection capabilities of the fluorescence imaging system.

Figure 2d shows the fluorescence image brightness uniformity testing area, which was composed of five 4 mm $\times$ 3 mm circular holes located at the center and at a 0.7 times field of view, as indicated in Fig. 1. These circular holes formed a test block for assessing fluorescence imaging distortion and the uniformity of the fluorescence image brightness.

Figure 2e shows the fusion accuracy testing area, featuring four L-shaped figures at the corners and eight circular blocks at various positions, which together formed the test block for evaluating the fusion accuracy. The four-corner L-shaped figures were designed to test whether the fluorescence image covers no less than 95% of the area of the color image, with the requirement that the tip of the angle of the L-shaped figure falls precisely at 0.95 of the maximum area of the color image. The other eight circular blocks, each with dimensions of 10 mm $\times$ 5 mm, were sunken and distributed at 0.25, 0.5, and 0.85 times the maximum field of view of the color image. These were used to test the fusion accuracy between the color and fluorescence images.

### Fluorescence image resolution, focal plane uniformity

**Fig. 3:**
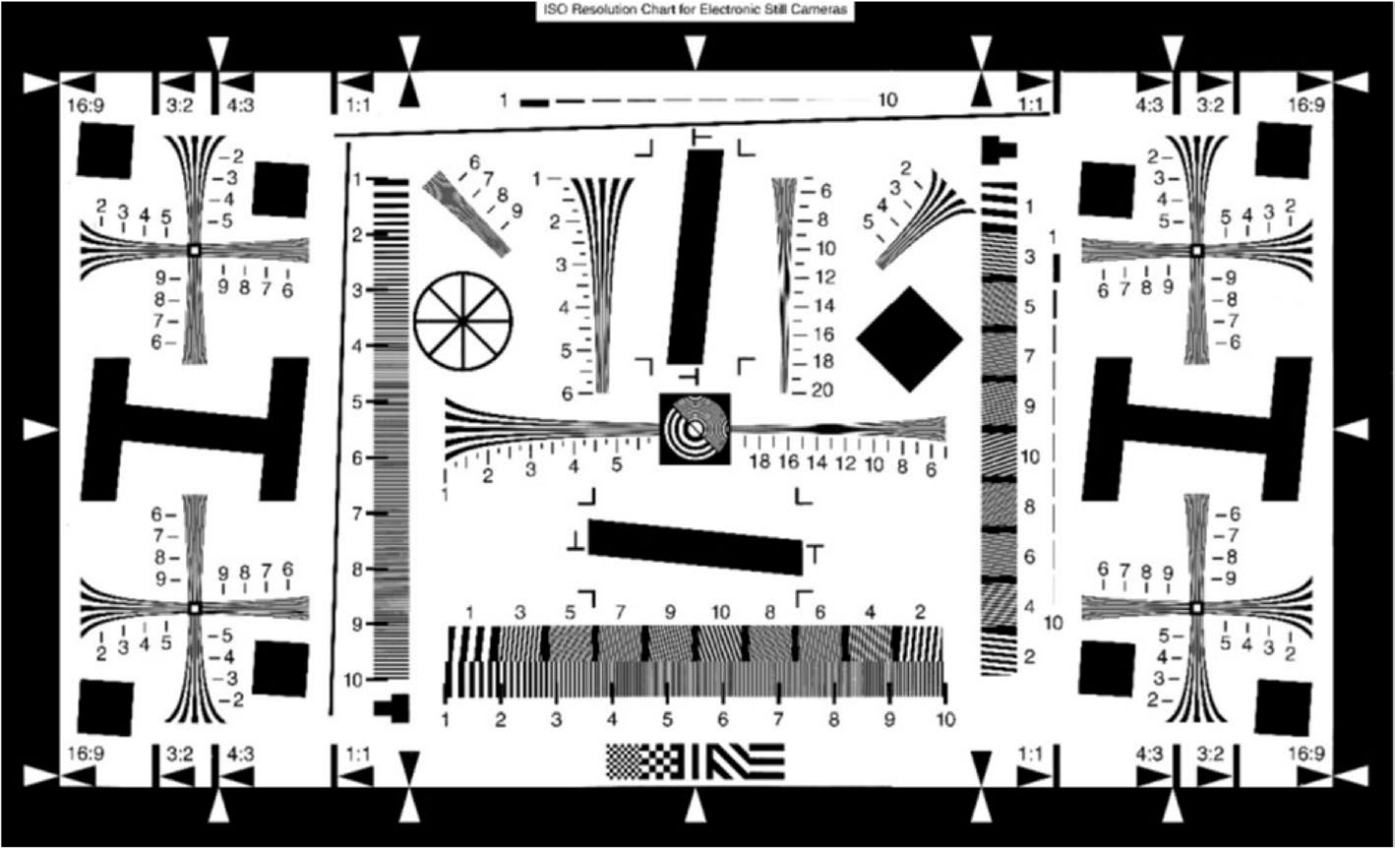
Resolution test card

The spatial resolution or sharpness of an imaging system is a critical metric for assessing the image quality and is inherently linked to the capacity of the system to discern fine details. In the realm of NIRF imaging, the USAF 1951 resolution test chart has been a prevalent choice for evaluation, according to previous research [32]. Building on this, we employed the transmission resolution test card specified by the ISO 12233 standard issued by the International Organization for Standardization (ISO) to assess the resolution capabilities of endoscopic systems quantitatively. This standard method serves as a benchmark for evaluating the performance of fluorescence endoscopic imaging systems. The test card, depicted in Fig. 3, features an effective area enclosed by a thick black border.

The assessment of focus plane uniformity is a crucial metric for evaluating image clarity and focusing quality, exerting a significant influence in domains such as autofocus systems, image processing, and optical design. The evaluation methodology for focus plane uniformity entails an integrated analysis of the images obtained throughout the entire field of view [33]. This process involves selecting suitable test subjects, capturing images, and using image processing techniques to assess the focus quality quantitatively. Within the scope of this research, we employed the resolutions of both fluorescence and white-light images to calculate the focus plane uniformity. The results derived from the following formula represent the uniformity of the focal plane:

1 $$\begin{aligned} \Delta d = \left|\frac{{{R_W} - {R_F}}}{{{R_F}}}\right| \end{aligned}$$

where $\Delta d$ is the focal plane uniformity, ${R_W}$ is the white-light image resolution, and ${R_F}$ is the fluorescence image resolution.

### Fluorescence signal-to-background ratio, sensitivity, and dynamic response range

In fluorescence imaging techniques, the SNR and signal-to-background ratio (SBR) are key metrics for evaluating the performance of an imaging system, and are critical for ensuring the image quality and accuracy of the analyzed results [34]. The SNR measures the ratio of the signal strength to background noise and reflects the ability of an imaging system to distinguish between signal and noise. A high SNR indicates that the signal is much higher than the noise level, allowing for more accurate detection and quantification of fluorescent signals. In fluorescence imaging, the SNR directly affects the ability to detect weak signals, that is, the fluorescence sensitivity.

SNR is the imaging SNR, *S* is the average signal intensity in the measured fluorescence region, and *N* is the standard deviation of the signal in the measured fluorescence region. The SBR measures the ratio of the signal intensity to the background intensity and reflects the ability of the imaging system to extract the signal from the background. A high SBR helps to identify fluorescently labeled areas more clearly and reduces the impact of background interference on the image analysis. The SBR is calculated using the following formula:

2 $$\begin{aligned} SBR = S/B \end{aligned}$$

where SBR is the imaging SBR, *S* is the average signal intensity of the measured fluorescence region, and *B* is the average signal intensity of the background region.

Fluorescence sensitivity usually refers to the ability of an imaging system to detect weak fluorescent signals. This indicator evaluates the response of a system to low concentrations of fluorescent markers or small signal changes. High fluorescence sensitivity means that the system can detect lower fluorescence intensities, thus playing a key role in applications such as biomarkers and the early diagnosis of diseases. In this system, the average signal gray value needs to be at least 30 to ensure that the lowest concentration of the fluorescent solution can be accurately detected; this concentration is defined as the fluorescence sensitivity (denoted as CL). The DR refers to the range of fluorescence signal intensities that can be distinguished by the imaging system and is usually expressed on a logarithmic scale. It ranges from the lowest detectable signal (fluorescence sensitivity) to the highest signal intensity that does not saturate. A high DR indicates that the system can simultaneously detect fluorescent signals ranging from very dark to very bright, which is critical for quantitative analysis and multi-marker imaging. When the average gray value of the fluorescent region is close to the saturation point, we identify it as the highest fluorescence concentration (denoted as CH). From this, we can calculate the fluorescence DR in decibels (dB).

3 $$\begin{aligned} DR = 20lg(CH/CL) \end{aligned}$$

### Fluorescence image brightness uniformity, fluorescence imaging aberration, and fluorescence resolution

Luminance uniformity reflects the consistency of the luminance distribution across the image field of view, implying that there are no significant local variations in the illumination or fluorescence signal intensity of the image across the field of view, which helps to prevent image interpretation errors caused by uneven luminance [35].

Experiments were conducted based on Fig. 2b, where images were captured and analyzed to measure the fluorescence average gray value of 90% of the area of the container hole diameter, with the image measurement at the center as $F_1$ and the image measurement around the edges as $F_2-F_5$. For fluorescence nudging, the fluorescence image luminance uniformity is the ratio of the average of the measurements around the edges to that at the center, *G*, with the following expression:

4 $$\begin{aligned} G = \frac{{\bar{F} }}{{{F_1}}} \end{aligned}$$

where $\bar{F}$ is the average of the edge fluorescence image measurements of $F_2-F_5$. Aberrations are nonlinear distortions that appear in an image and can be caused by aberrations in the optical system, irregularities in the shape of the sample, or curvature of the imaging plane. They can cause straight lines in an image to appear curved or cause the actual size and shape of an object to not match the imaging results. Controlling and correcting aberrations are critical for maintaining image accuracy and performing precise measurements.

The radial dimensions of the diameter of the center circular spot ($D_0$) and diameters of the four surrounding circular spots ($D_i,i=1,2,3,4$) were measured and recorded separately, which was repeated three times.

A single measurement of the unit relative aberration ${V_{U - \infty }}$ is denoted by ${V_{U - \infty ,i}}$ ($i=1,2,3,4$) and the calculation is given by

5 $$\begin{aligned} {V_{U - \infty ,i}} = \frac{{{D_0} - {D_i}}}{{{D_0}}} \end{aligned}$$

The unit relative aberration of the *Z* field of view can be calculated from the unit relative aberration using the following equation:

6 $$\begin{aligned} {V_{U - z}} = \frac{{\sum \limits _i^4 {{V_{U - \infty ,i}}} }}{4} \end{aligned}$$

The measurement process was repeated three times, the arithmetic mean of the measurement results was obtained, and the unit relative aberration consistency was calculated.

7 $$\begin{aligned} {U_V}_{rel} = \mathrm{max} \left|\frac{{{V_{U - z,i}} - {V_{U - z,j}}}}{{2{V_{U - z}}}}\right| \end{aligned}$$

When taking absolute values,

8 $$\begin{aligned} {U_V} = \mathrm{max} |{V_{U - z,i}} - {V_{U - z,j}}|/2 \end{aligned}$$

The final results are expressed as the arithmetic mean of the difference in agreement between the three measurements.

Fluorescence resolution is a measure of the ability of an imaging system to distinguish between adjacent objects or structures, which directly affects the clarity of the image and the visualization of details. It is usually defined in terms of the smallest distance that can be distinguished. A higher resolving power means that the system can capture finer structural features, which is critical for applications such as viewing fluorescent images and intracavitary tissues. Computer software was used to determine the limiting resolution distance (mm) of the sector line of the standard mimic resolution region, and the resulting resolution was the fluorescence resolution.

### Fluorescence penetration depth

The fluorescence penetration depth is a key parameter in fluorescence imaging that determines the ability of an imaging technique to penetrate deep into biological tissues or materials. This metric is important for enabling three-dimensional visualization of deep tissue structures, performing multispectral imaging analyses and quantitative fluorescence concentration measurements, guiding clinical diagnosis and surgical navigation, and evaluating the internal properties of materials in materials science. The depth of fluorescence penetration is influenced by the optical properties of the fluorescent marker, optical absorption and scattering properties of the sample, and wavelength of light used. Therefore, careful consideration is required in the design of fluorescence endoscopes to ensure the accuracy and depth of imaging.

The fluorescence test mimic was the same as that shown in Fig. 2d. There were seven horizontal slots with different gradients in the detection depth region of the fluorescence test mimic. The bar groove in the middle of each gradient was the location for placing the fluorescent solution bar, and the center of each adjacent bar groove was equidistant from the center of the bar; the fluorescent solution bar (3 mm outside diameter) was covered with a fat emulsion solution (intralipid 10%). Each step represents a different depth gradient indicated by *h*, which is the distance from the upper surface of each step to the liquid surface of the fat emulsion solution. The image acquired in this area was analyzed, and the fluorescence gray value of the fluorescent solution rod area was measured when the number of imaging frames was not less than 60 and the SNR was not less than 10 B, as described above. The average signal gray value should not be less than 30, and the corresponding maximum value was the fluorescence detection depth.

### Fluorescence fusion accuracy

Fluorescence image fusion accuracy ensures that information from different fluorescent channels is accurately represented in the synthesized image, thus providing a more comprehensive view of the sample. Moreover, high-precision image fusion enables researchers to observe and interpret the interactions and relative positions of different fluorescent markers more clearly, thereby contributing to an in-depth understanding of biological processes. If multiple fluorescent markers are used in an experiment, accurate image fusion ensures that the signals of each marker are correctly recorded and differentiated, thereby optimizing the experimental design. Therefore, fluorescence image fusion accuracy is a key factor that affects fluorescence images in fluorescence endoscopic photography systems.

The fluorescence test mimic was the same as that in Fig. 2e, and there were four L-shaped grooves in the corners and eight fusion accuracy test holes in the fusion accuracy area of the fluorescence test mimic. First, it was necessary to ensure that the frame rates of the white-light and fluorescence images were the same and perform a time synchronization test, followed by a field of view range test, and finally, the fusion shooting of white-light and fluorescence images was completed. The pixel coordinates of the center of the circle of the white-light color image are defined as $({X_{i1}},{Y_{i1}})$, and the centroid pixel coordinates of the fluorescent image are defined as

$$\begin{aligned} ({X_{i2}},{Y_{i2}}) \end{aligned}$$

*i* is defined as the number of sampling times and must be greater than 3. The fusion accuracy is calculated as follows:

9 $$\begin{aligned} \begin{array}{l} J{X_i} = |{X_{i2}} - {X_{i1}}|,\quad \mathrm{ }JX = \max J{X_i}\\ J{Y_i} = |{Y_{i2}} - {Y_{i1}}|,\quad \mathrm{ }JY = \max J{Y_i} \end{array} \end{aligned}$$

where $J{X_i}$ is the *x*-axis image fusion accuracy calculated using the *ith* sampling, *JX* is the *x*-axis image fusion accuracy, $J{Y_i}$ is the *y*-axis image fusion accuracy calculated using the *ith* sampling, and *JY* is the *y*-axis image fusion accuracy.

## Results

### Image resolution and focal plane uniformity experimental results

**Fig. 4:**
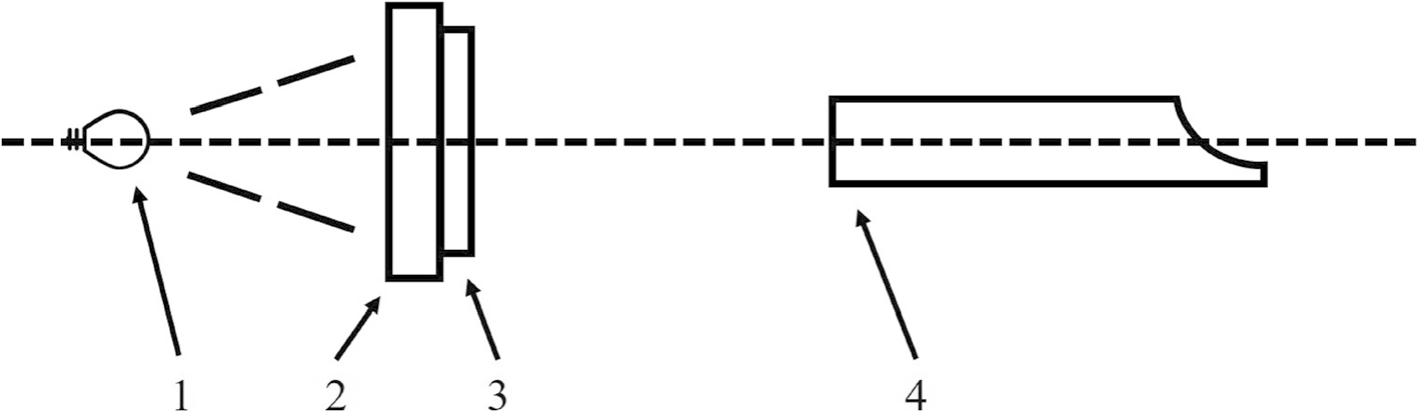
Schematic of the resolution test, where 1 is the test light source, 2 is the homogenizing device, 3 is the test plate, and 4 is the camera system

A uniform device was placed in front of the test light source with a wavelength range of 850 nm $\pm$ 30 nm so that the light was sufficient and uniformly irradiated in the resolution test card, and the illumination difference was less than 10%. The positions of the test light source, resolution test card, and lens are shown in Fig. 4; the resolution test card was perpendicular to the lens axis of view so that the center of the resolution test card was aligned with the center of the lens, and the length and width of the resolution test card were aligned with the horizontal and vertical directions of the lens screen, respectively. The corresponding effective area in the resolution test card was selected according to the aspect ratio that could be captured by the camera system, and the distance between the camera system lens and resolution test card was adjusted such that the effective area of the resolution test card filled the field of view. After the focus became clear, image acquisition was performed. Computer software was used to determine the limiting clear resolution of the wedge line in the J2 or K2 regions of the resolution test card, and the resolution result obtained was the fluorescence resolution. The value calculated by the system was 1400 lines.

**Fig. 5:**
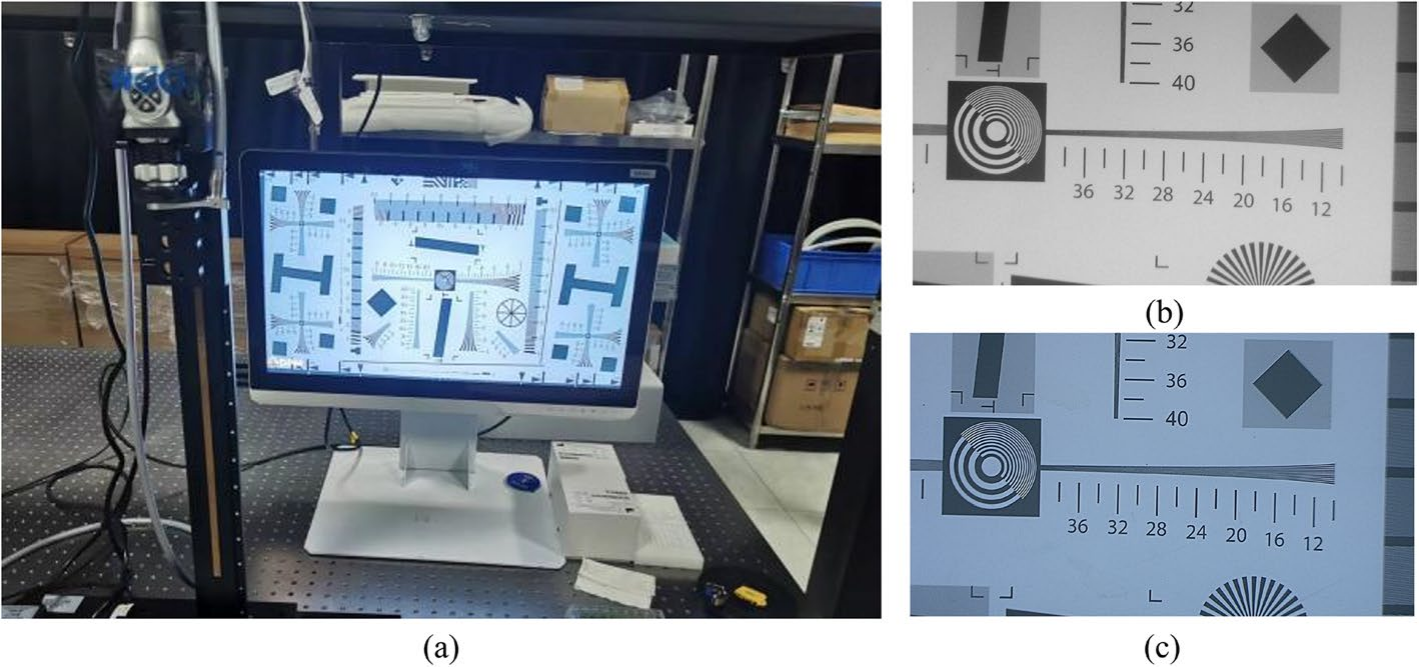
Resolution test experimental procedure. **a** The overall image of the resolution test card; **b** a fluorescence resolution image; and **c** a white-light resolution image

The camera system was moved to an optically designed working distance and only the white-light band of the test light source was turned on. After the focus became clear, image acquisition was performed. The results are shown in Fig. 5. The test card wedge line limit resolution was visually determined and this value was recorded for the color image resolution, which was the white-light image resolution. At this time, the value of 2200 lines was determined. Subsequently, according to Eq. 1, the focal plane uniformity of the system was 0.57.

### Experimental results of fluorescence SBR, sensitivity, and DR

**Fig. 6:**
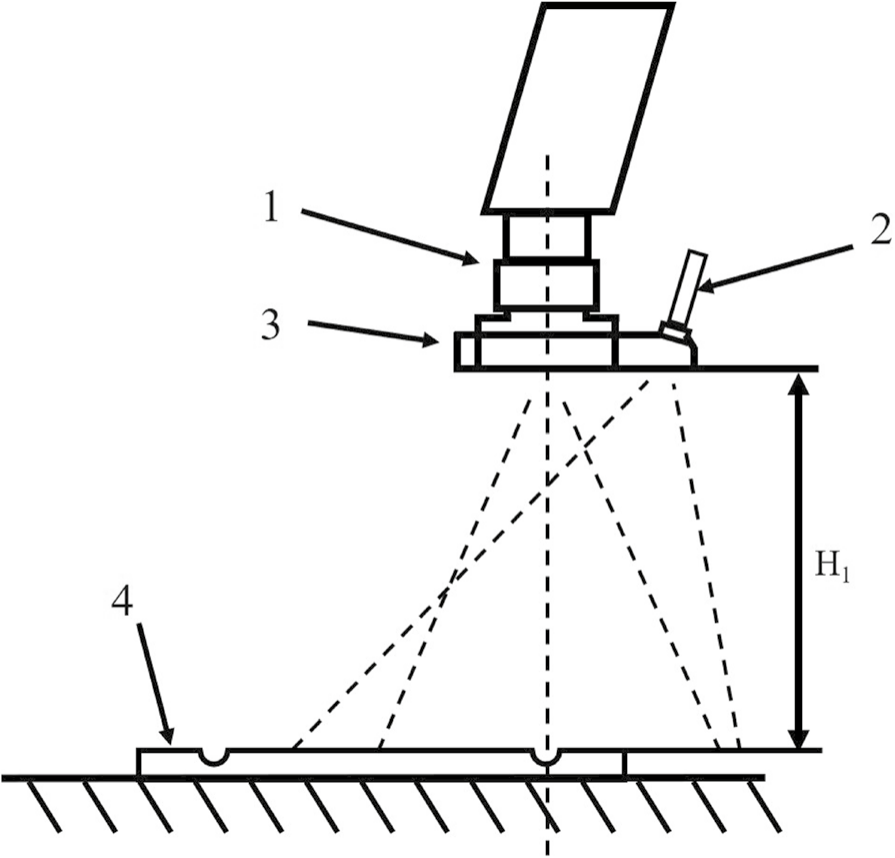
Schematic of the light-guide beam fixing tooling, where 1 is the camera system, 2 is the light-guide beam, 3 is the connection tooling, and 4 is the fluorescence test simulator

Using the connection tooling, the light-guide beam and camera were mounted together, and the architecture is shown in Fig. 6. The center of the light-guide beam out of the light end face and the center of the lens end cap face to the imaging surface were the same distance, with a tolerance of ± 2 mm. The distance between the center of the light-guide beam out of the light end face and the center of the lens end cap face was A *leq* 50 mm, and the center of the light-guide beam illumination overlapped with the center of the imaging field of view on the imaging surface.

The fluorescent solution for testing was prepared with a two-fold gradient dilution method, using 1 mL ICG solution with 1 mL serum as the base solution, and the dilution of the fluorescent solution in other wells was configured according to the above method. We obtained 260 $\normalsize \mu L$ of the configured fluorescent solution in the order of concentration, and dropped it into the fluorescent test well plate container position in sequence, keeping the liquid surface level with the surface of the well plate.

As shown in Fig. 6, the height of the camera was adjusted so that the distance H1 from the lower face of the camera to the fluorescence test hole plate was 150 mm. The position of the container holes in the fluorescence test hole plate was moved so that the position of the container holes of the fluorescent solution of the concentration to be tested was in the center of the field of view. The fluorescence effect of the container holes of the various concentrations was observed individually, and images were collected. A stepwise normalization process was implemented on the acquired images according to the process in Fig. 2a. To ensure that the number of imaging frames was not less than 60 and that the SNR reached at least 10 B, we measured the fluorescence gray value of the region with 90% of the vessel pore diameter.

**Fig. 7:**
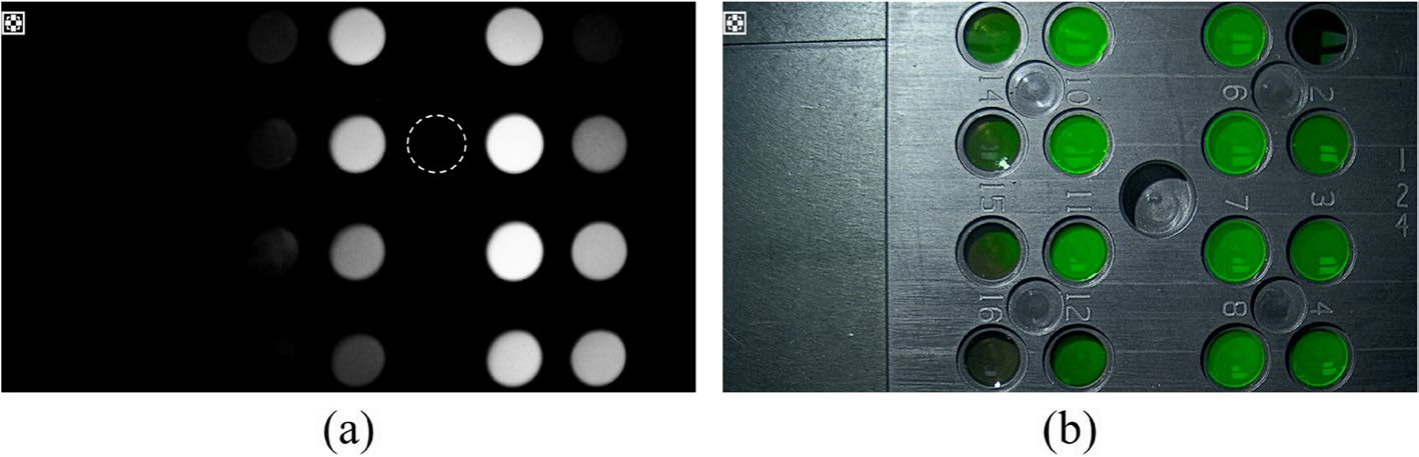
Fluorescent SBR, sensitivity, and DR result images. **a** Fluorescence mode image; **b** fluorescence fusion image

In this experimental assessment, we designated the circular region outlined by the dashed line in Fig. 7 as the background area and selected an area adjacent to the current test hole. The bright area corresponded to a concentric circle encompassing approximately 80% of the area of each test hole. The average signal intensity of the fluorescence region, denoted as *S*, for the brightest test hole was 241.80. Within this functional area, there were 16 test holes of varying concentrations, exhibiting gray values ranging from 10 to 241. The standard deviation of the signal in the fluorescence region, denoted as *N*, was 24.66, whereas the average signal intensity in the background region, denoted as *B*, was 1.046. After calculating the average signal intensity *S*, standard deviation *N* of the fluorescence region, and average signal intensity *B* of the background region using software, we employed Eq. 2 to determine the SNR of the fluorescence image, which was 19.83. Furthermore, using Eq. 3, we calculated the fluorescence image SBR of the system to be 253. Subsequently, the lowest fluorescent solution concentration CL that could be detected was 0.153 $\normalsize \mu g/mL$, and the highest fluorescent concentration CH at the saturation point of the average gray value was 4.883 $\normalsize \mu g/mL$, which could be calculated according to Eq. 4 as DR = 20*lg(CH /CL) = 20*lg(4.883/0.153) = 30.08 dB.

### Experimental results of fluorescence image brightness uniformity, fluorescence imaging aberration, and fluorescence resolution

**Fig. 8:**
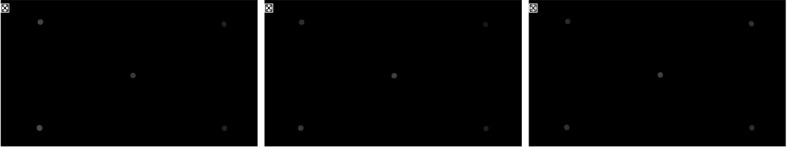
Results of three samples of endoscopic fluoroscopy systems

The fluorescence test mimic was placed in the same position as in Fig. 6. The height of the lens was adjusted so that the edge of the mimic coincided with the maximum field of view, and then focused clearly. The fluorescent solution with the highest fluorescence concentration (the concentration corresponding to the CH value) was pipetted and dropped into the five holes of the image brightness uniformity test block of the fluorescence test mimic, with the liquid level flush with the upper surface of the holes, and the images were captured. The results are shown in Fig. 8. The collected images were analyzed to measure the fluorescence average gray value of the 90% area of the diameter of the container holes and to calculate the fluorescence average gray value of the image measurements at the center and the image measurements around the edges. The results are shown in Table 1. The fluorescence image luminance uniformity *G* of the system was calculated to be 90.5%.

**Table 1:** Fluorescence average gray value

	$F_1$	$F_2$	$F_3$	$F_4$	$F_5$	$\bar{F}$
Gray value	169.60	152.00	158.8	158.80	144.60	153.55

Subsequently, the radial dimensions of the diameter of the center circular spot ($D_0$) and diameters of the four surrounding circular spots ($D_i,i=1,2,3,4$) were measured and recorded separately, which was repeated three times. The result are shown in Table 2. The fluorescence imaging aberrations were calculated, and the results yielded a unit relative aberration $V_{u-z}$ = 12.3% for the *Z* field of view and an overall fluorescence imaging aberration parameter $U_v$ = 2.3% for the system.

**Table 2:** Circular spot diameter

Number	$D_0$	$D_1$	$D_2$	$D_3$	$D_4$
1	23 mm	21 mm	22 mm	22 mm	20 mm
2	23 mm	21 mm	22 mm	22 mm	20.5 mm
3	23 mm	21 mm	21 mm	22 mm	21 mm

**Fig. 9:**
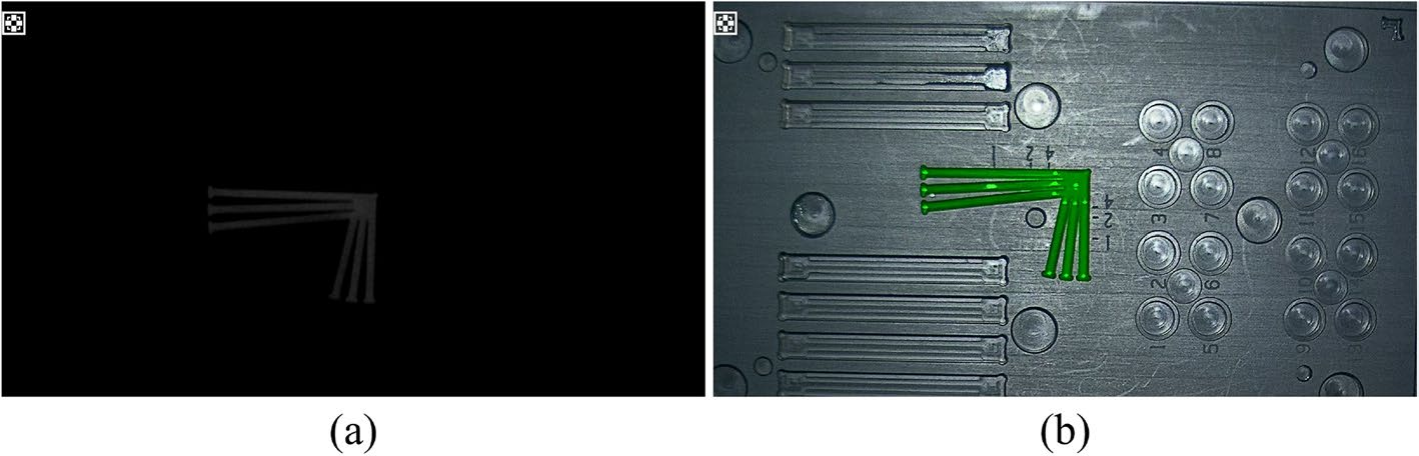
Fluorescence resolution result image. **a** Fluorescence pattern image; **b** fluorescence fusion image

A drop of the fluorescent solution with the same concentration as the highest fluorescent concentration was pipetted into the linear groove of the fluorescent resolution test area of the fluorescent test mimic, with the level of the liquid flush with the upper surface of the mimic. Without changing the height of the lens, the mimic body was moved to use the resolution test area in the center of the field of view of the lens area, and image acquisition was performed, as shown in Fig. 9. Computer software was used to determine the standard mimic body resolving the power area sector line limit resolution distance (mm) and the minimum spacing of distinguishable neighboring straight lines was less than 0.25 mm. Therefore, the fluorescence resolving power index of the system was 0.25 mm.

### Results of fluorescence penetration depth experiments

**Fig. 10:**
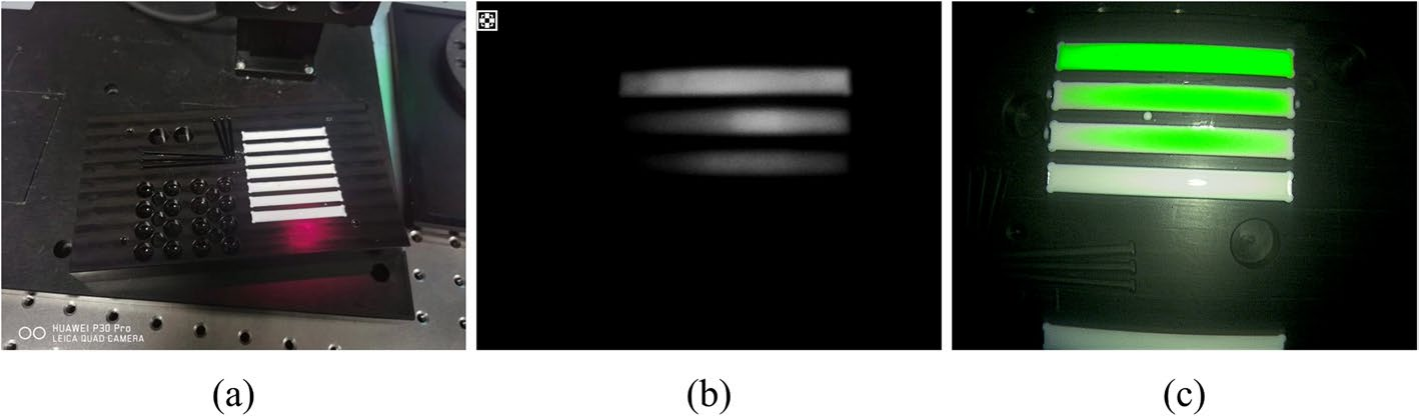
Fluorescence penetration depth test results. **a** The experimental process image; **b** the fluorescence pattern image; and **c** the fluorescence fusion image

The fluorescence test mimic was the same as that in Fig. 1. There were seven horizontal slots with different gradients at the depth of the detection region of the fluorescence test mimic. The bar slots in the middle of each gradient were the locations where the fluorescent solution bars were placed, and each adjacent bar slot was equally spaced from the center of the bar. The fluorescent solution bars (3 mm outside diameter) were covered with a fat emulsion solution (intralipid 10%), with each step representing a different depth gradient, denoted by *h*, which was the distance from the upper surface of each step to the liquid surface of the fat emulsion solution. The same test platform as that shown in Fig. 6 was used, where the distance from the lens end cap surface of the camera system to the liquid surface of the fat emulsion solution was the common working distance. The fluorescent solution bar was placed in the step bar groove of the measured depth, and the fluorescence test mimic was moved so that the solution bar was in the center of the field of view. The depth position of the fluorescent solution bar was replaced one by one, and the above steps were repeated to observe the fluorescence image of each depth step, as shown in Fig. 10. The collected images were analyzed and the fluorescence gray value of the fluorescent solution bar area was measured. The depth of detection was 3.5 mm, 4.5 mm, and 5.5 mm from top to bottom. The test result was a fluorescence detection depth of no less than 5.5 mm.

### Results of fluorescence fusion accuracy experiments

First, the white-light and fluorescence images were detected to have the same frame rate, after which a stopwatch was placed in the field of view and the monitor was set to display the white-light and fluorescence images on a split screen. Using a camera or video camera, photos/videos of the white-light and fluorescent images were displayed on the monitor simultaneously. The difference between the stopwatch readings of the white-light and fluorescent images in the same photo/video frame image was examined, which should be less than 20 ms. The test results are shown in Table 3. Passing the test led to proceeding to the next test and vice versa for failing, and the fusion accuracy was out of specification.

**Table 3:** Time synchronization test results

Number	Watch time	Display time	Delay time
1	3m:41s:69cs	3m:41s:59cs	100 ms
2	3m:43s:83cs	3m:43s:73cs	100 ms
3	3m:48s:09cs	3m:47s:99cs	100 ms
4	3m:53s:99cs	3m:53s:93cs	60 ms
5	3m:53s:49cs	3m:53s:43cs	60 ms

**Fig. 11:**
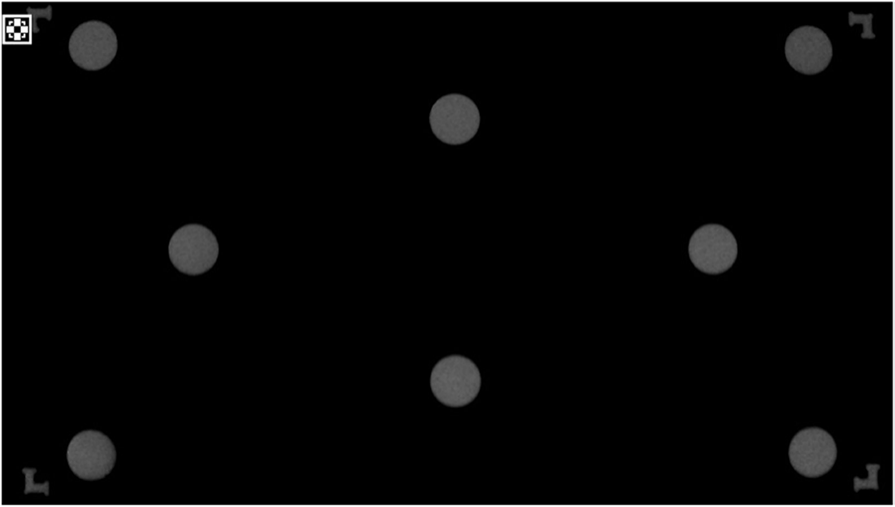
Fluorescence imaging field of view difference test passed with white-light imaging field of view difference test passed

After meeting the requirements of the time synchronization test, the fluorescent solution with the highest fluorescence concentration (the corresponding concentration of the CH value) was transferred to the four L-shaped grooves and eight test holes in the fusion accuracy test area of the fluorescence test simulator, and the liquid surface was flush with the upper surface of the holes. According to the test schematic shown in Fig. 6, the lens of the camera system was fixed on top of the fluorescence test mimic, which was adjusted to image clearly under white light, and the edge of the fluorescence test mimic was flush with the edge of the maximum field of view of the white light. The lens was maintained motionless and the fluorescence mode was turned on. In addition, it was determined whether the fluorescence image of the four L-shaped grooves at the corners of the fluorescence test simulator were all complete and visible, and that the image was not missing at the corners or other parts. The results are shown in Fig. 11. If the image was intact, the difference between the fluorescence imaging field of view and the white-light imaging field of view passed the test, and the next test could be performed.

**Fig. 12:**
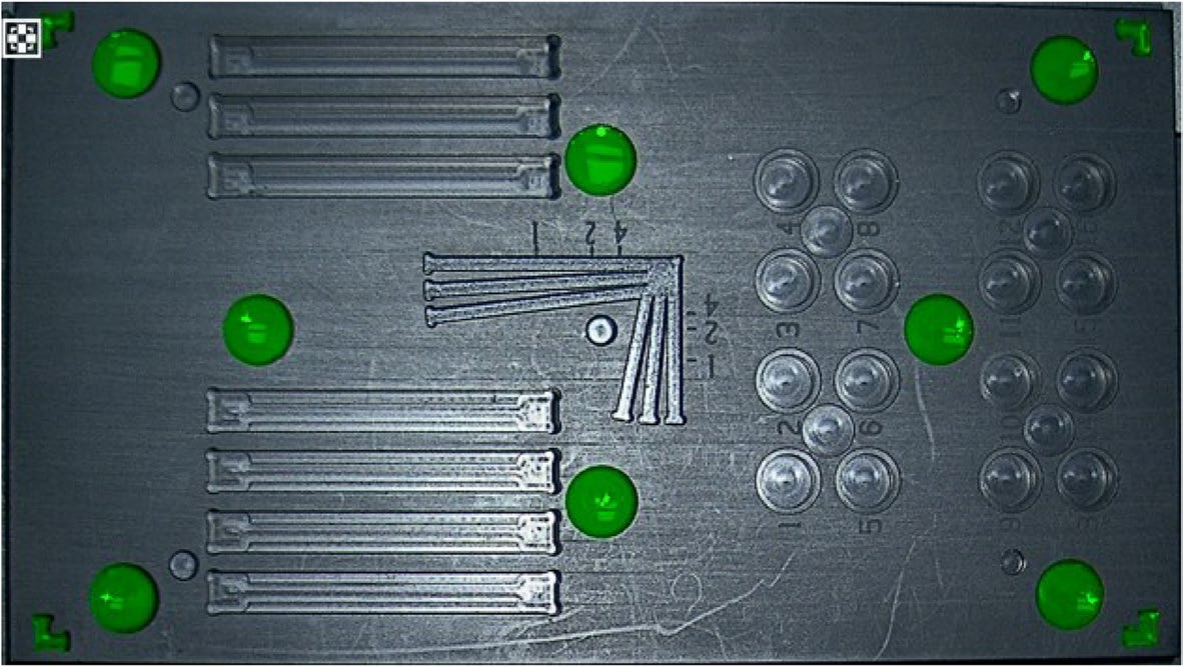
Fluorescence test mimic fusion accuracy test region for fluorescence fusion image results

Subsequently, white-light color images were obtained, following which the lens was kept still, the white-light camera was turned off, the fluorescence camera was turned on, fluorescence images were obtained, and the fusion of fluorescence and white-light images was performed. The fused image is shown in Fig. 12, and the fusion accuracy was calculated. The fusion accuracy of the *x*-axis image was calculated to be JX = 7 and that of the *y*-axis image was calculated to be JY = 5.

## Discussion

This study proposes a set of technical criteria for evaluating the image quality of fluorescence endoscopic photography systems with the aim of improving the performance of imaging systems and the accuracy of clinical applications. These criteria are theoretically instructive; however, there are some potential shortcomings in their practical application. First, the testing conditions may be too ideal and fail to simulate the complexity of the clinical environment adequately, which may affect the generalizability of the standards. Second, the rapid development of technology means that existing standards must be updated continuously to accommodate emerging technologies and methods. In addition, despite our emphasis on the importance of standardization, the implementation of these standards in different healthcare settings may be challenged by differences in equipment and operators. Factors such as subjective differences in user operations, cost-effectiveness considerations, and individual patient differences may also affect the effectiveness of standards implementation. An inadequate assessment of long-term stability and reliability may limit the understanding of how devices are used in actual clinical settings. Specialty requirements for data interpretation and analysis may limit the involvement of a wider range of healthcare professionals. Finally, translating these standards from laboratory settings into clinical practice may face multiple barriers such as regulations, training, and workflow integration. Future research should focus on these shortcomings and explore solutions to ensure that fluorescence endoscopic photography systems can provide high-quality imaging in diverse clinical settings, while maintaining cost-effectiveness and ease of operation. In addition, further research should focus on improving the flexibility and adaptability of standards, as well as developing more advanced imaging techniques and analytical tools to meet changing clinical needs.

## Conclusions

This study has thoroughly explored the standardized requirements that must be met by fluorescence endoscopy equipment before achieving widespread clinical application. It clarified the requirements for cold light sources containing NIR excitation light and provided detailed experimental methods to ensure the stable and reliable performance of the cold light source. This study sets forth clear requirements for various performance indicators of NIRF photography systems and provides the corresponding experimental methods to assess and ensure the performance of the imaging system. It stipulates the evaluation criteria and experimental methods for the image quality of medical endoscopy imaging systems, ensuring that the image quality meets clinical needs. To compare clinical outcomes effectively, assess the performance of imaging systems, and accurately quantify fluorescence signals during fluorescence endoscopy, it is crucial to establish unified standardization protocols.

Although the test methods in standards may not be appropriate for all medical devices with fluorescence technology, the study can serve as a guide for establishing performance evaluation tasks throughout the device life cycle, including design input, design output, product validation and verification, and clinical trial standardization. In addition, these methods may not be adaptable for novel system designs, but the standards in this study could be adopted in other medical observation devices with fluorescent imaging capabilities based on practicality.

In the realm of technical evaluation, multiple processes, such as preclinical assessment, clinical evaluation, and registration testing, are often performed to evaluate the security and effectiveness of medical devices. Various methods can be used to assess the fluorescence imaging quality, including the use of mimetic bodies, biological sample testing, animal trials, and other innovative approaches. The insights presented in this study offer an evaluation method for fluorescent endoscopic medical devices that can also be utilized to establish standardized research protocols.

## Data Availability

All data included in this study are available upon request by contact with the corresponding author Yu An.

## Abbreviations

LED: Light-emitting diode
NIR: Near-infrared
IEC: International Electrotechnical Commission
AORN: Association of periOperative Registered Nurses
NIRF: Near-infrared fluorescence
SNR: Signal-to-noise ratio
ISO: International Organization for Standardization
SBR: Signal-to-background ratio
DR: Dynamic range
